# Graphene oxide and mineralized collagen-functionalized dental implant abutment with effective soft tissue seal and romotely repeatable photodisinfection

**DOI:** 10.1093/rb/rbac024

**Published:** 2022-04-29

**Authors:** Yichun Gao, Ke Kang, Bin Luo, Xiaoqing Sun, Fang Lan, Jing He, Yao Wu

**Affiliations:** National Engineering Research Center for Biomaterials, Sichuan University, Chengdu 610064, P.R. China

**Keywords:** dental implant abutment, soft tissue sealing, antibacterial, graphene oxide, mineralized collagen

## Abstract

Grasping the boundary of antibacterial function may be better for the sealing of soft tissue around dental implant abutment. Inspired by ‘overdone is worse than undone’, we prepared a sandwich-structured dental implant coating on the percutaneous part using graphene oxide (GO) wrapped under mineralized collagen. Our unique coating structure ensured the high photothermal conversion capability and good photothermal stability of GO. The prepared coating not only achieved suitable inhibition on colonizing bacteria growth of *Streptococcus sanguinis*, *Fusobacterium nucleatum* and *Porphyromonas gingivalis* but also disrupted the wall/membrane permeability of free bacteria. Further enhancements on the antibacterial property were generally observed through the additional incorporation of dimethylaminododecyl methacrylate. Additionally, the coating with sandwich structure significantly enhanced the adhesion, cytoskeleton organization and proliferation of human gingival fibroblasts, which was effective to improve soft tissue sealing. Furthermore, cell viability was preserved when cells and bacteria were cultivated in the same environment by a coculture assay. This was attributed to the sandwich structure and mineralized collagen as the outmost layer, which would protect tissue cells from photothermal therapy and GO, as well as accelerate the recovery of cell activity. Overall, the coating design would provide a useful alternative method for dental implant abutment surface modification and functionalization.

## Graphical Abstract

**Figure:**
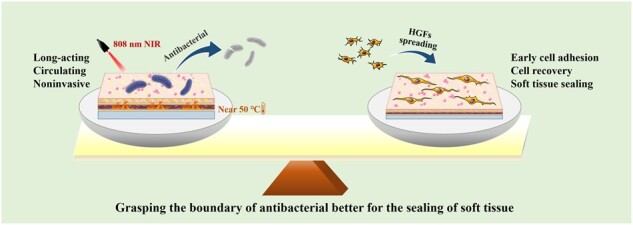

## Introduction

Dental implant infection, especially around the percutaneous part, has remained a serious clinical problem [1]. Accordingly, much more attention has been devoted to the development of antibacterial materials to defend against infections. However, another important issue that needs to be considered in biomaterial design is the sealing ability of the soft tissue around the implant abutment [1]. Unfortunately, most of the introduction of antibacterial components into the dental implant abutment could adversely affect the cells around dental implants and damage their biological functions [2]. Designing materials that meet both antibacterial and soft tissue sealing abilities has been a challenging task.

For instance, altering the physicochemical properties of the substrate, including polyethylene glycol [3] and polyhexamethylene biguanide brushes [4], has widespread recognition owing to the reduction of bacterial adhesion. This ‘passive’ mode of antibacterial presents promising bioactivities, but the effectiveness of these coatings for reducing bacterial adhesion is very limited and varies greatly depending on bacterial species [5]. Another strategy is based on the introduction of antibacterial metal ions, including Ag^+^, Cu^2+^ and so on [6, 7], called ‘active’ antibacterial. However, there have been numerous reports on adverse effects on the cell response associated with high concentrations [8]. Compared with other implants, higher antibacterial demands, including long term, on-demand and cycling-used antibacterial properties, are put forward for the development and design of dental implants abutment [9], due to the hostile and diverse microbial environment of an oral cavity, which could contain up to 600 different bacterial species [10] and be subject to infectious disease such as peri-implantitis [11].

Light-assisted photothermal therapy has received a great deal of attention in the field of non-invasive treatment due to its advantages, including negligible invasiveness, reduced side effects, and highly effective bacteria-killing capability [12]. Near-infrared (NIR) light killing bacteria in the way of induced local hyperthermia depends on photothermal conversion agents, including molybdenum disulfide (MoS_2_) [13], black phosphorus [14], graphene oxide (GO) [15] and MXene [16]. As a common photothermal conversion agent, GO is known for low cost, easy synthesis, easy modification and good photothermal conversion performance [17–20]. For instance, Cui *et al.* fabricated GO-coated PEEK implant through polydopamine (PDA). Although the coating showed good antibacterial efficacy, the biocompatibility of the modified PEEK implant was significantly reduced possible due to the direct interaction between GO and tissue cells [21]. However, while GO-modified coatings can effectively enhance the antibacterial properties, the light-induced hyperthermia could also damage the normal cells and healthy tissues around the implants [22], leading to impairment of bioactivity. Moreover, GO with sharp edges can directly target bacterial membranes, disrupting membrane permeability [23, 24]. Meanwhile, this unique structure would inevitably slice through and penetrate the membrane, resulting in disrupting normal cell functions [25]. Thus, it is necessary to develop a novel strategy to guarantee the biocompatible character of the GO-based materials while possessing excellent antibacterial properties.

**Figure 1.**
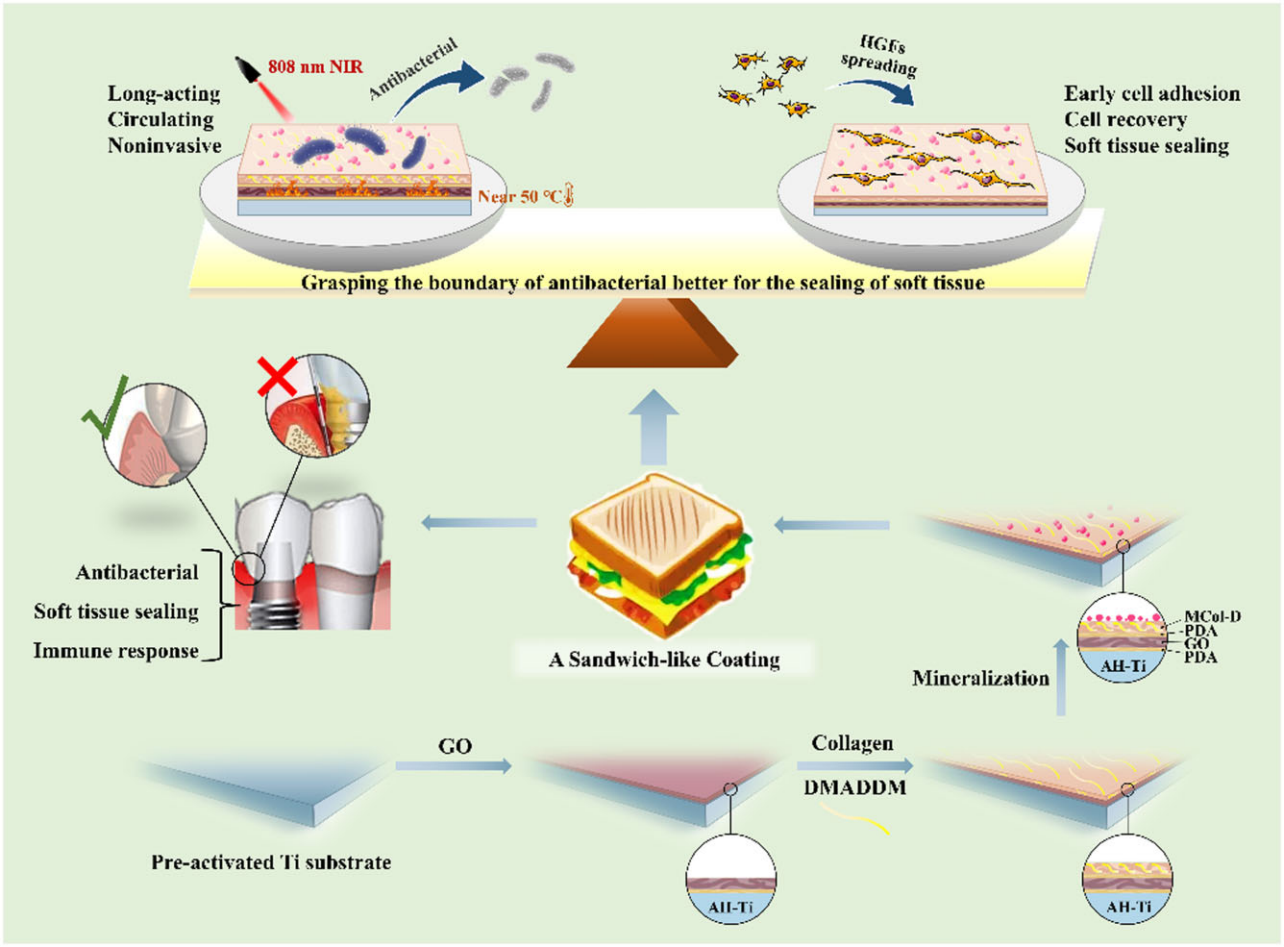
Schematic illustration of the preparation of the sandwich-structured dental implant abutment coating using GO wrapped under mineralized collagen to achieve a controllable antibacterial property based on suitable sealing of soft tissue

To address this challenge, we designed and fabricated a sandwich-structured dental implant abutment coating using GO wrapped under collagen. In our system, inducting the dopamine can not only implement the robust combination of GO and Ti alloy but also contribute to the enhancement of photothermal effect of GO. Collagen as the outmost layer supports the soft tissue sealing due to its good biocompatibility and further mineralization. Additionally, collagen layer can be as a carrier for antibacterial substances, including antimicrobial peptides and quaternary ammonium salt, to achieve multimodal antibacterial activities. We envisage that the as-obtained coating system would have combined advantages from its constituent materials and unique structure, as well as have features of a controllable antibacterial efficiency based on steady and long-term sealing of soft tissue (Fig. 1).

## Materials and methods

### Material preparation

#### Bioactivation of titanium substrate by alkaline-heat treatment

Commercial pure Ti plates with a thickness of 1.5 mm, a diameter of 14 mm and a purity of 99.5% were provided by Northwest Institute for Nonferrous Metal Research. Before alkali-heat (AH) treatment, the Ti substrate was polished with 240, 400, 600, 800, 1000, 1200 and 1500 grid sandpaper and then washed by distilled water, acetone, ethyl alcohol and distilled water for 30 min, respectively [26]. Afterwards, the cleaned Ti substrates were immersed in NaOH solution (5 mol/l) for 24 h at 60°C. Finally, the substrates were heated up to 600°C at a rate of 5°C/min and cooled to room temperature after keeping warm in muffle furnace for 1 h [27].

#### Fabrication of Ti-GO-MCol and Ti-GO-MCol-D

We fabricated Ti-GO-MCol and Ti-GO-MCol-D by layer-by-layer technology. Briefly, the preparation was conducted as follows. Firstly, dopamine (5 mg/ml, Sigma-Aldrich) was selected to coat the preactivated Ti substrates as previous reports [26]. During the 24-h incubation period, the PDA layer was formed. Secondly, the GO solution (3 mg/ml in 30% ethanol solution) was dropped on the aforementioned substrate. After evaporation at 60°C for 1 h, the GO layer was uniformly and stably deposited on titanium plate through electrostatic incorporation, which was called Ti-PDA-GO. Thirdly, after soaked in dopamine solution (2 mg/ml) and shaken for 12 h, we prepared the second layer of PDA coating to provide a platform for collagen grafting. Then, the substrate was immersed in collagen solution with or without dimethylaminododecyl methacrylate (DMADDM, 5 mg/ml, State Key Laboratory of Oral Diseases of Sichuan University) overnight at 4°C. Finally, the prepared substrate was vertically immersed in 15 ml 1.5 × simulated body fluid (1.5 × SBF) and mineralized for 24 h at 37°C. The two kinds of coatings were referred as Ti-GO-MCol and Ti-GO-MCol-D, respectively.

### Coating characterizations

The preparation process of the coating was characterized by Raman spectroscopy (RM, LabRAM HR, HORIBA, French) with 633 nm light for excitation. Fourier transform infrared spectroscopy (FTIR, UV3600, Shimazu, Japan) was used to analyze the chemical functional group of the sample surfaces. Surface morphology and composition were observed by field emission scanning electron microscopy (FE-SEM, S-4800, Hitachi, Japan) and energy dispersive spectroscopy (EDS, Phenom, Philips, Nederland). Atomic force microscopy (AFM, MFP-3D-BIO, Asylum Research, USA) was used to examine the topography of the PT-Ti, Ti-MCol, Ti-GO-MCol and Ti-GO-MCol-D, respectively. In addition, the wettability was also examined by the water contact angle analysis (CA, Easy Drop, KRUSS, Germany).

### Photothermal effects

The photothermal abilities of all the samples, including PT-Ti, Ti-MCol, Ti-GO-MCol, Ti-GO-MCol-D, Ti-PDA, Ti-PDA-GO and Ti-PDA-GO-PDA, were characterized with an infrared thermal imager. Briefly, different samples (14 mm in diameter) were placed in a 24-well culture plate under wet condition (1 ml, phosphate buffer solution, PBS) and then were irradiated with an 808 nm laser for 10 min at different laser power densities (0.5, 1.0, 1.5 W/cm^2^). And the temperature values and thermal images were recorded every minute.

### Antibacterial capacity

#### Bacteria culture

*Streptococcus* *sanguinis* (SK1), *Fusobacteriumnucleatum* (ATCC 25586) and *Porphyromonasgingivalis* (ATCC 33277), the common oral bacteria, were provided by State Key Laboratory of Oral Diseases of Sichuan University and used in the antibacterial tests. *Streptococcussanguinis* was cultivated in brain heart infusion (BHI) while *F. nucleatum* and *P. gingivalis* were cultivated in BHI and BHI agar with 1% hemin and 1% vitamin K under strictly anaerobic conditions with 80% N_2_, 10% H_2_ and 10% CO_2_ at 37°C.

#### Bacterial inhibition

The bacteria resuspended at a density of 1 × 10^8^ CFUs/ml were spread on the surfaces of PT-Ti, Ti-MCol, Ti-GO-MCol and Ti-GO-MCol-D samples in a 24-well plate. After incubated for 24 h, the samples were irradiated with NIR light (1 W/cm^2^) for 15 min. The 3-(4,5-dimethylthiazol-2-yl)-2,5-diphenyl tetrazolium bromide (MTT, J&K Scientific, Beijing, China) assay was used to evaluate the antibacterial ability of the materials and determined by optical density (OD) values. The morphology of the three kinds of bacteria was observed by SEM after treated with 2.5% glutaraldehyde at 4°C overnight and dehydrated in a series of ethanol concentration gradient of 30, 50, 70, 90, 100 and 100 v/v %, respectively.

#### Circulating antibacterial ability

The circulating antibacterial ability of Ti-GO-MCol and Ti-GO-MCol-D was investigated. Briefly, 1 ml of diluted bacterial suspension (1 × 10^8^ CFUs/ml) was added to the surface of Ti-GO-MCol and Ti-GO-MCol-D. Subsequently, the samples were illuminated at 0, 8, 24, 48 and 72 h, respectively, and continued to be cultivated for 72 h. The MTT assay was used to test the ability of circulating antibacteria by means of OD values.

### Human gingival fibroblast response

#### Cell culture

Primary human gingival fibroblasts (HGFs) were obtained from the State Key Laboratory of Oral Diseases (Sichuan University, Chengdu, China). HGFs were cultured in Dulbecco’s Modified Eagle’s Medium (DMEM, high glucose) supplemented with 10% fetal bovine serum, 100 μg/ml streptomycin and 100 U/ml penicillin and kept in an incubator at 37°C with 5% CO_2_. HGFs were passaged every 2 days and passages of 2–10 were used for the experiments.

#### Cell proliferation

In order to evaluate cell proliferation, the cell counting kit-8 (CCK-8, Dojindo, Kumamoto, Japan) assay was conducted. HGFs at a density of 5 × 10^4^ cells/well were seeded on each sample in a 24-well plate. After 1, 3, 5 and 7 days of culture, the CCK-8 assay was used in accordance with the instructions and the OD values at 450 nm were recorded.

#### Cell adhesion

HGFs were seeded on different samples in 24-well plate and the cell count was adjusted to 5 × 10^4^ cells/well. After 1 and 4 h of incubation, viable cells were labeled with fluorescein diacetate (FDA), whereas dead ones were stained with propidium iodide.

Besides, in order to explore the effect of NIR light on cell adhesion and spread, HGFs were cultured on Ti-GO-MCol and Ti-GO-MCol-D for 24 h and then the samples were irradiated with 808 nm NIR laser (1 W/cm^2^) for 15 min. Afterwards, the cells were stained with FDA and analyzed using a laser confocal scanning microscope (CLSM, TCS SP5, Leica, Germany).

#### Immuno-fluorescence staining of adhesion-related proteins

Suspension of HGFs with the density of 2 × 10^4^ cells/well was seeded on the PT-Ti, Ti-MCol, Ti-GO-MCol and Ti-GO-MCol-D samples, respectively. After cultured for 24 h, immunofluorescence stainings of vinculin and integrin β1 were used to analyze cell adhesion and spread. Briefly, HGFs cultured on the samples were fixed with 4% paraformaldehyde for 30 min at room temperature. Then, 0.1% Triton X-100 was used to permeabilize cell membranes for 10 min at 4°C. After blocked by 4% bovine serum albumin for 30 min at 37°C, the cells were stained with an anti-vinculin (dilution at 1:50, rabbit anti-vinculin, Abcam) and integrin β1 (dilution with a concentration of 10 μg/ml, mouse anti-integrin β1, Abcam), followed by DyLight 488-conjugated anti-mouse IgG antibody (dilution at 1:200, Abcam) and anti-Rabbit IgG (dilution at 1:200, Abcam), respectively. Finally, phalloidin Alexa 594 was used to visualize the actin cytoskeleton and 2-(4-amidinophenyl)-6-indolecarbamidine dihydrochloride (DAPI) was stained for the nucleus. All samples were observed using CLSM (LSM800, Zeiss, Germany).

In order to examine the effect of NIR light on the above-specified proteins (vinculin and integrin β1), the experiment was split into two parts. The cells were cultured on Ti-GO-MCol and Ti-GO-MCol-D for 24 h. Subsequently, one part was directly used for CLSM observation after irradiated with 808 nm NIR laser (1 W/cm^2^) for 15 min, while the other part continued in culture for further 24 h in an incubator at 37°C with 5% CO_2_ before confocal microscopy.

### Effect of the coatings on membrane permeability

#### Permeability of the *S. sanguinis*, *F. nucleatum* and *P. gingivalis* wall/membrane

Similar to the bacteria inhibition studies in Bacterial inhibition section, 1 ml of suspension of the *S. sanguinis*, *F. nucleatum* and *P. gingivalis* (with an initial bacterial density of 1 × 10^8^ CFUs/ml) were spread onto PT-Ti, Ti-MCol, Ti-GO-MCol and Ti-GO-MCol-D samples with empty well plate as control. Before and after NIR irradiation, glucose-6-phosphate dehydrogenase (G6PDH) activity that was collected from precipitation after centrifugation was measured according to the instructions (S0189, Beyotime, China). The leakage of nucleic acid from supernatant after centrifugation was also detected by ultramicro spectrophotometer (NANODROP 2000, Thermo Scientific, USA) at 260 nm.

#### Permeability of the HGF cell membrane

Membrane permeability of HGFs was determined by the leakage level of lactate dehydrogenase (LDH) in the culture medium as a result of disruption of cell membrane. HGFs were seeded on PT-Ti, Ti-MCol, Ti-GO-MCol and Ti-GO-MCol-D samples loaded in a 24-well plate at a density of 1 × 10^5^ cells/well for 24 h. Then, LDH analysis was performed according to the manufacturer's instructions of LDH Cytotoxicity Assay Kit (C0016, Beyotime, China).

Meanwhile, we also examined the effect of NIR light on membrane permeability through the LDH release from the cells on Ti-GO-MCol and Ti-GO-MCol-D after illumination. Besides, in order to evaluate the effect of our materials on the recovery of cell membrane, after the above treatments, the samples were continually cultured in the incubator for a further 24 h and LDH analysis was carried out again. Additionally, fluorescent membrane dye PKH26 (PKH26GL-1KT, Sigma-Aldrich, USA) was used to stain the cells to measure membrane permeability and membrane recovery.

### Coculture of HGFs and bacteria

In order to explore the antibacterial properties of the coatings under the condition of coculture of HGFs and bacteria, 1 ml bacterial suspension with a density of 1 × 10^8^ CFUs/ml per well was added on the surfaces of the sterilized PT-Ti, Ti-MCol, Ti-GO-MCol, Ti-GO-MCol-D, Ti-GO-MCol-NIR and Ti-GO-MCol-D-NIR. Then, all the samples were cultivated under strictly anaerobic conditions at 37°C for 60 min to allow adherence of the bacteria to the material surface. Afterwards, HGFs were diluted with coculture medium (DMEM containing 2% BHI without streptomycin and penicillin) to a concentration of 5 × 10^4^ cells/ml and then 1 ml cells/well was added. After 4 h of coculture at 37°C with 5% CO_2_, cell cytoskeleton was stained with phalloidin Alexa 594 and the nucleus were stained with DAPI. The morphology of cell and bacteria was observed by SEM. In order to examine the effect of NIR light, the Ti-GO-MCol-NIR and Ti-GO-MCol-D-NIR were illuminated immediately with bacterial suspension, and the samples were performed as described above.

### Statistical analysis

All results were statistically analyzed by SPSS Software (SPSS Inc., Chicago, IL, USA). Differences between more than two treatment groups were determined using one-way analysis of variance followed by Fisher's least significant difference test. *P*-values <0.05 were considered significant. All experimental data were represented as means with standard errors (mean ± SE). All quantitative data were independently repeated three times unless otherwise stated.

## Results and discussion

### Surface characterization

#### Surface fabrication

**Figure 2.**
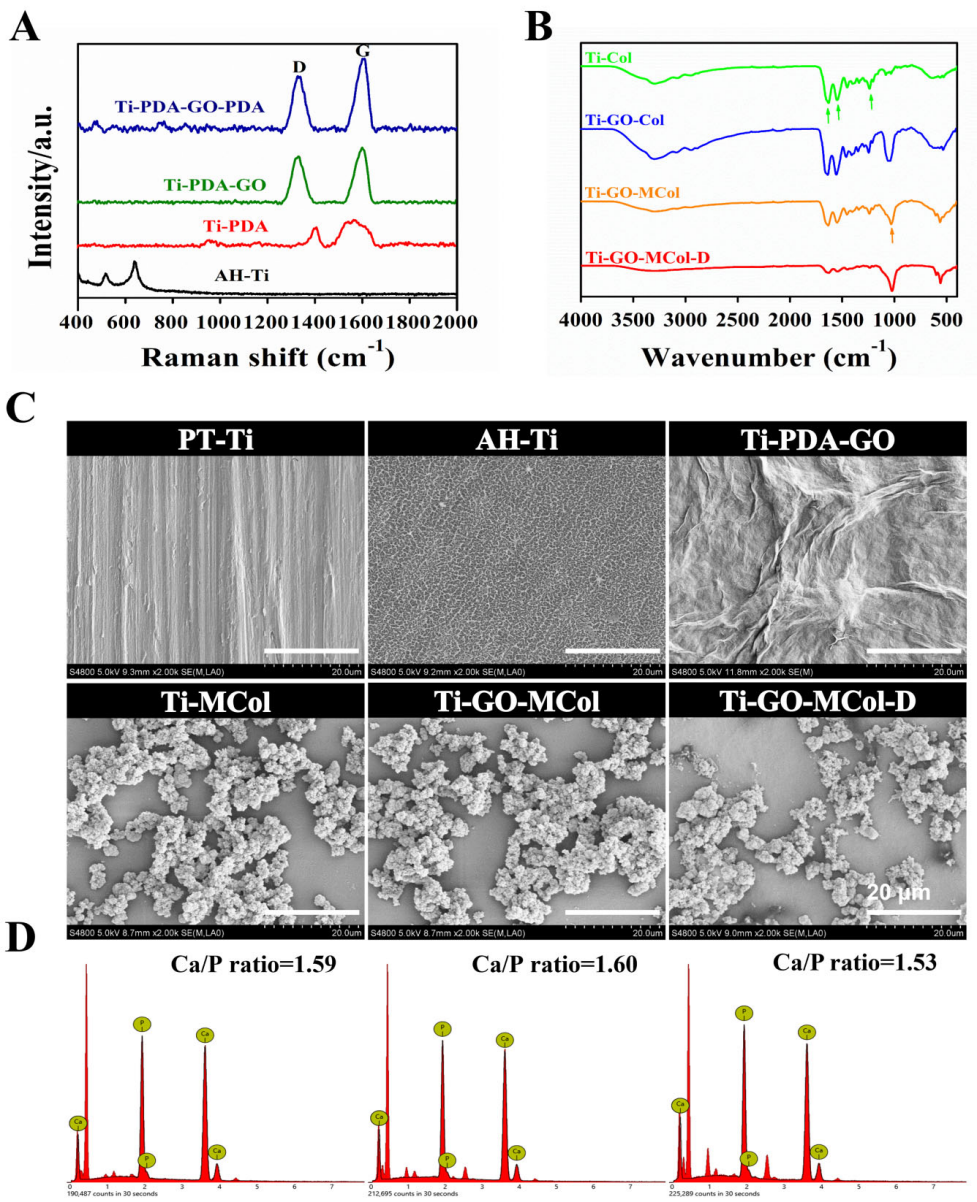
(**A**) Raman spectra of AH-Ti, Ti-PDA, Ti-PDA-GO and Ti-PDA-GO-PDA. (**B**) FTIR spectrum of Ti-Col, Ti-GO-Col, Ti-GO-MCol and Ti-GO-MCol-D. (**C**) SEM images of PT-Ti, AH-Ti, Ti-PDA-GO, Ti-MCol, Ti-GO-MCol and Ti-GO-MCol-D. Scale bars represent 20 μm. (**D**) EDX results of Ti-MCol, Ti-GO-MCol and Ti-GO-MCol-D

The preparation process of Ti-GO-MCol-D was shown in Fig. 2. As shown in Fig. 2A, Raman spectra for AH-Ti indicated that the preactivation was successful after AH treatment, based on the presence of the peaks at 515 and 640 cm^−1^ corresponding to anatase and rutile, respectively [28, 29]. After the first layer of PDA was polymerized, new peaks appeared at 1360 and 1580 cm^−1^, which were the characteristic peaks of deformation of catechol and stretching vibration in PDA structure [30]. As the PDA had covered the surface of AH-Ti, the characteristic peak of AH-Ti disappeared, indicating that the PDA layer was successfully prepared. When GO layer appeared, the Raman spectrum showed characteristic peaks of the D and G bands at 1335 and 1600 cm^−1^, respectively [31]. After the self-polymerization of the second layer of PDA, the intensity ratios of the D/G peaks decreased, indicating that the disorder of the material decreased [32]. This may be due to the successful grafting of PDA, which restored the defect of GO. Moreover, the toxicity of GO correlated well with their extent of surface oxidation. The lower the oxidation degree of GO, the less toxic it was [33]. The grafting of PDA on the GO surfaces increased amination, which could reduce the oxidation degree of GO [34].

Figure 2B showed the FTIR spectra of the Ti-PDA-GO-PDA coatings after collagen graft. Three peaks of 1624, 1545 and 1236 cm^−1^ can be observed in Ti-GO-Col coatings, corresponding to amide I, amide II and amide III bands of type I collagen [35], indicating that the collagen layer was successfully grafted. Moreover, after mineralization, the strength of amide II and amide III bands of collagen decreased significantly while the amide I band redshifted slightly. This might be due to the preferential coordination between free calcium ions and carbonyl groups in collagen and gradual mineralization [36]. Meanwhile, a new peak of 1030 cm^−1^ appeared, which was designated as the peak of ${\text{PO}}_{4}^{3-}$, due to the formation of Ca-P minerals [37]. The peak of ${\text{PO}}_{4}^{3-}$ could still be observed on Ti-GO-MCol-D, indicating that the addition of DMADDM did not affect the mineralization of collagen. However, the stretching vibration absorption peak of carbon–oxygen double bond in ester group of DMADDM (1724 cm^−1^) was hardly visible as compared with Ti-GO-Col-D (Supplementary Fig. S1) [38]. From the above results, we have successfully prepared a coating similar to ‘sandwich’ structure connected by dopamine, where GO was wrapped under the mineralized collagen layer.

#### Surface morphology

Figure 2C showed the morphologies of the coating surfaces at different stages of surface modification. The as-prepared Ti surface exhibited a very smooth topography with visible polishing lines. After preactivation, a prickly porous structure was formed, which was a typical feature after AH treatment. The GO addition significantly altered the Ti-PDA-GO surface morphology, showing wrinkled and corrugated structure. After the presence of collagen layer, it appeared that collagen had well coated on the prepared substrate and the surface was smooth and homogenous, indicating that GO was successfully wrapped under the collagen layer (Supplementary Fig. S2). It was also observed that apatite crystallites were deposited on the collagen layers due to the mineralization. The EDX results indicated that elements Ca and P were present in the agglomerated particles and the Ca/P ratio was about 1.60 (Fig. 2D). Importantly, apatite crystallites displayed a dispersed distribution, benefited from our method for controlled mineralization time [39]. The sparse distributions of apatite crystallites favored collagen exposure that provided cell adhesion site and facilitated the work of DMADDM. Moreover, the presence of GO and DMADDM did not significantly alter the morphology and distribution of apatite crystallites.

#### Surface roughness

**Figure 3.**
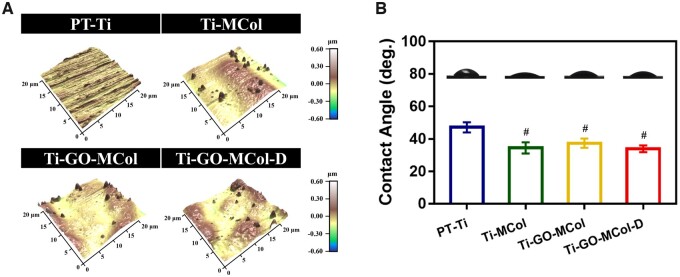
(**A**) Atom force microscopy images of PT-Ti, Ti-MCol, Ti-GO-MCol and Ti-GO-MCol-D. (**B**) Water contact angle of PT-Ti, Ti-MCol, Ti-GO-MCol and Ti-GO-MCol-D. Error bars represent standard deviation of the mean for *n* = 4 (^#^*P* < 0.05 compared with PT-Ti)

In the case of modifications used for dental implants abutment, surface roughness is an important surface parameter. As shown in Fig. 3A and Supplementary Table S1, AFM results demonstrated that the roughness of PT-Ti after polishing was about 45.37 ± 2.55 nm. After modification, the surface roughness value of Ti-GO-MCol increased to 97.13 ± 1.36 nm, probably due to the formation of flat collagen film and appropriate self-mineralization. Moreover, the roughness value of Ti-GO-MCol-D was about 95.99 ± 2.71 nm, indicated that the addition of DMADDM did not obviously influence the surface roughness. As reported, the surface roughness of dental implants abutment should be <200 nm which, to date, is regarded as a ‘gold standard’ [40]. A smooth surface with a certain roughness is conducive to cell adhesion, but when the surface roughness exceeds the threshold of 200 nm, it would be conducive to bacterial adhesion and encourage the formation of biofilm [41]. The overall values of these samples did not exceed 200 nm, which met the requirement of clinical use.

#### Surface wettability

In addition to roughness, water contact angle is also important to dental implants abutment. As it was shown in Fig. 3B, the water contact angle of PT-Ti was 49.64 ± 0.36 degrees. After surface modification, considerable reduction in the contact angle was noticed with Ti-MCol, Ti-GO-MCol and Ti-GO-MCol-D loading to 30.82 ± 1.76, 35.12 ± 0.34 and 35.34 ± 1.20 degrees, respectively. In general, the hydrophilic surface was more conducive to cell adhesion [42], and the hydrophilic surfaces with water contact angle of 20–40 degrees were better [43].

### Photothermal effect

**Figure 4.**
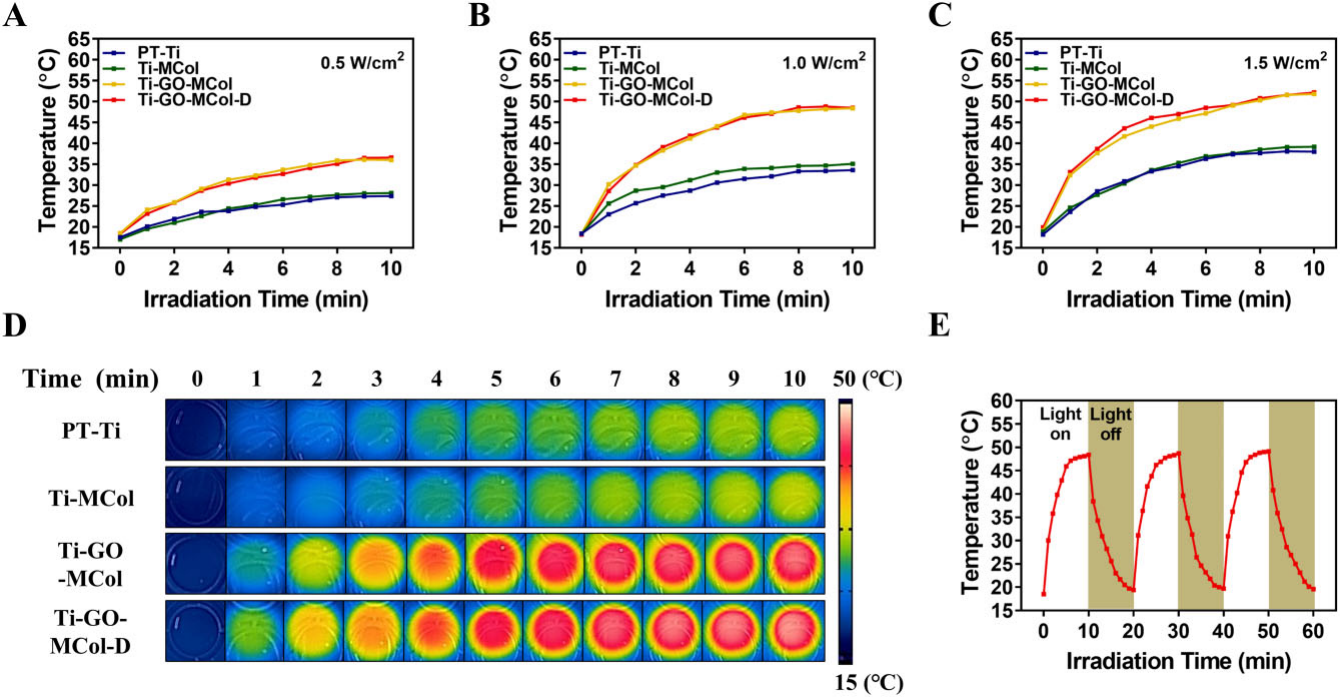
(**A–C**). Photothermal curves of PT-Ti, Ti-MCol, Ti-GO-MCol and Ti-GO-MCol-D under 808 nm laser irradiation of different power density (0.5, 1.0 and 1.5 W/cm^2^). (**D**) Infrared thermal images of PT-Ti, Ti-MCol, Ti-GO-MCol and Ti-GO-MCol-D under 808 nm laser irradiation of 1.0 W/cm^2^ power density. (**E**) Heating curves of Ti-GO-MCol-D for three laser on/off cycles (808 nm, 1 W/cm^2^)

The addition of antibacterial component GO makes it necessary to investigate the photothermal effect of our coatings. To evaluate the photothermal performance of different samples, PT-Ti, Ti-MCol, Ti-GO-MCol and Ti-GO-MCol-D were treated with 808 nm laser irradiation (0.5, 1.0 and 1.5 W/cm^2^), respectively. Figure 4A showed that when the samples were treated with NIR light with power density of 0.5 W/cm^2^ for 10 min, the temperature of PT-Ti and Ti-MCol, which were without GO coating changed very slightly. The final temperature of the two groups was basically the same, which was about 25°C. After the preparation of the GO coating, the final temperature of Ti-GO-MCol and Ti-GO-MCol-D changed obviously, which raised to about 40°C after the 10 min of irradiation. But it was not enough for antibacteria [44]. After increasing the power density of the NIR light from 0.5 W/cm^2^ to 1.0 and 1.5 W/cm^2^, the final temperature of Ti-GO-MCol could rise to about 47°C and 53°C, respectively (Figs. 4B and C). The addition of DMADDM also had no significant effect to the photothermal property of the coatings. Considering the possible damage to the cells bringing by photothermal effect, we finally chose the power density of 1.0 W/cm^2^ and the irradiation time of 10 min as the final experimental conditions (Fig. 4D). The infrared thermal images further indicated that compared with Ti-PDA-GO (Supplementary Figs. S3 and S4), the presence of collagen layers did not harm the photothermal effect, whereas it contributed to the increased photothermal temperature. This may be due to the addition of PDA in the process of collagen grafting. Previous studies demonstrated that the π-π stacking of PDA and GO further improved the photothermal property of GO [32]. Moreover, our results also indicated that the photothermal temperature of Ti-PDA-GO-PDA was higher than that of Ti-PDA-GO for any irradiation time. It was noteworthy that there was almost no change of photothermal performance post three times ON/OFF laser cycles, showing the high photothermal stability of Ti-GO-MCol-D (Fig. 4E). Overall, our coating with ‘sandwich’ structure could increase the photothermal effect.

### Antibacterial activity

**Figure 5.**
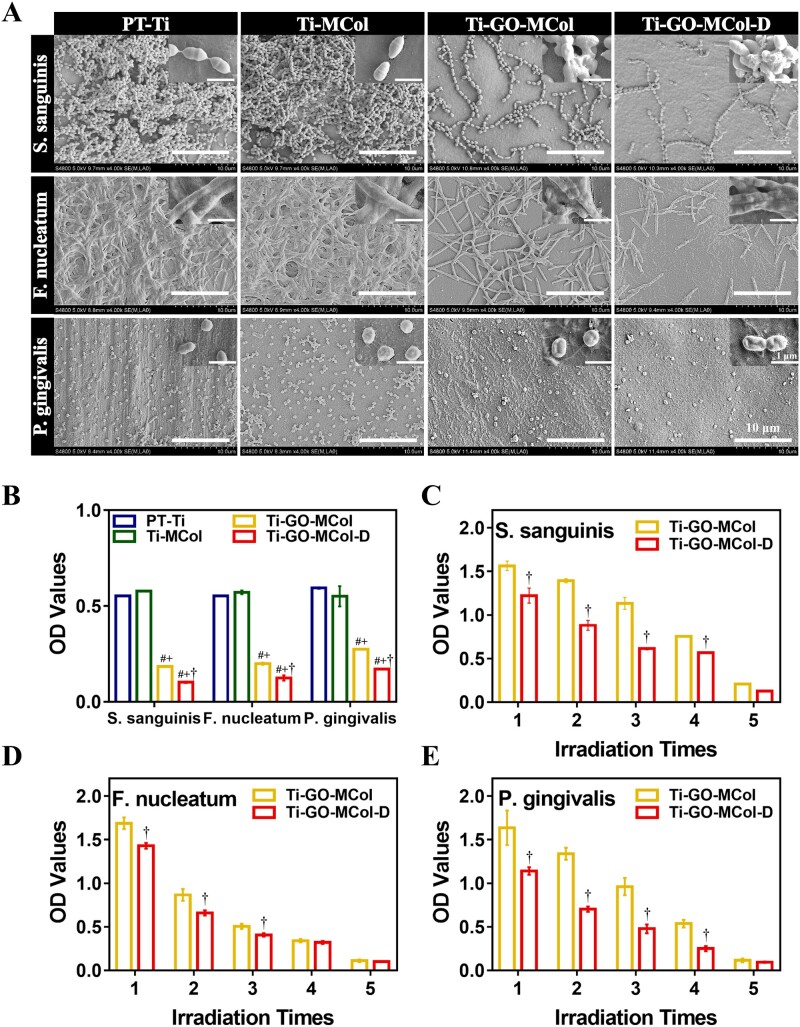
(**A**) SEM images of *S. sanguinis*, *F. nucleatum* and *P. gingivalis* on PT-Ti, Ti-MCol, Ti-GO-MCol and Ti-GO-MCol-D after illumination. Scale bars represent 10 μm. (**B**) MTT assay of *S. sanguinis*, *F. nucleatum* and *P. gingivalis* cultured on PT-Ti, Ti-MCol, Ti-GO-MCol and Ti-GO-MCol-D after illumination. (**C–E**). Cyclic antibacterial results of *S. sanguinis*, *F. nucleatum* and *P. gingivalis* on Ti-GO-MCol and Ti-GO-MCol-D after illumination from first to fifth cycle. Error bars represent standard deviation of the mean for *n* = 3 (^#^*P* < 0.05 compared with PT-Ti, ^+^*P* < 0.05 compared with Ti-MCol, ^†^*P* < 0.05 compared with Ti-GO-MCol)

First, we selected *S. sanguinis*, *F. nucleatum* and *P. gingivalis*, three different kinds of periodontal specific bacteria in the oral, to examine the antibacterial activity of the modified coatings. As shown in Fig. 5A, the *S. sanguinis* on the PT-Ti and Ti-MCol gathered together to form big clusters, suggesting positive conditions for the growth of *S.sanguinis*. However, for the Ti-GO-MCol after illumination, the number of visible bacteria had decreased substantially. This could be attributed to the addition of GO, where the temperature would rise under the irradiation of 808 nm NIR laser and bring some damage to bacteria. For the additional usage of DMADDM, a very scattered distribution of bacteria was found on the surface of Ti-GO-MCol-D, because DMADDM itself had good contact antibacterial ability as a cationic antibacterial agent. The enlarged picture showed that the bacterial cell membrane was destroyed. As for Gram-negative bacteria, *P. gingivalis* and *F. nucleatum*, the GO-modified coatings still had excellent antibacterial potential, reducing the extent of bacterial adhesion to low numbers. Moreover, synergistic antibacterial effect of DMADDM was also observed. The additional usage of DMADDM caused serious damage to the Gram-negative bacterial membrane, which the membrane of *F. nucleatum* disrupted with irregular and wrinkled morphology and the disruption of *P. gingivalis* membrane was observed with the presence of collapsing.

Figure 5B showed the quantitative number of bacteria. For all the three bacteria, the OD values of PT-Ti and Ti-MCol were not different, showing that the two samples did not have the ability to resist bacteria. When GO was introduced, the OD values of the samples decreased significantly, indicating that the temperature change caused by photothermal effect had great impact on bacteria. At the same time, it was not difficult to find that the sample added with cationic antibacterial agent DMADDM had the lowest OD values and showed the best antibacterial ability, confirming that DMADDM had good synergistic antibacterial ability with GO.

Circulating antibacterial ability is beneficial for promising practical treatment of disease, especially for dental implant infection. With this reproducible function can be prevented reinfection, without additional surgery or treatment. As shown in Figs. 5C–E, for all the three bacteria, as the number of photothermal cycle increased, the OD values of Ti-GO-MCol decreased, indicating that our GO-modified coating had excellent circulating antibacterial ability. After five cycles, Ti-GO-MCol showed more than 6.5-, 6.9- and 13.3-fold lifts in antibacterial effect relative to one photothermal cycle for *S. sanguinis*, *F. nucleatum* and *P. gingivalis*, respectively. Moreover, we found that synergistic effect of DMADDM on circulating antibacterial ability remained. For the first to the fourth cycles, the OD values of Ti-GO-MCol-D were significantly lower than that of Ti-GO-MCol, except that no significant difference was observed for *F. nucleatum*. It was not surprising that there were no significant differences between Ti-GO-MCol-D and Ti-GO-MCol in the fifth cycle, since almost all bacteria were dead. Overall, the above results confirmed that the presence of GO and DMADDM in the coating resulted in enhanced antibacterial effects against both the Gram-positive and Gram-negative bacteria. Furthermore, the antibacterial effects of these two active compounds were found to act synergistically. DMADDM has a direct capable of directly killing the bacteria like *S. sanguinis*, *F. nucleatum* and *P. gingivalis*, which can quickly kill bacteria as well as prevent bacterial colonization, thus avoiding the opportunity for heavily infected development. More importantly, GO could not only provide photothermal-induced early bactericidal activity but also treat reinfection without additional surgery or treatment via photothermal cycle. Besides, our results indicated that the temperature of Ti-GO-MCol and Ti-GO-MCol-D under NIR irradiation ramped quickly to 50°C after the 10 days of PBS immersion, indicating that our modified coatings provided the long term and recyclable use (Supplementary Fig. S5).

### Effect of modified coatings on the wall/membrane permeability of colonizing bacteria and free bacteria

**Figure 6.**
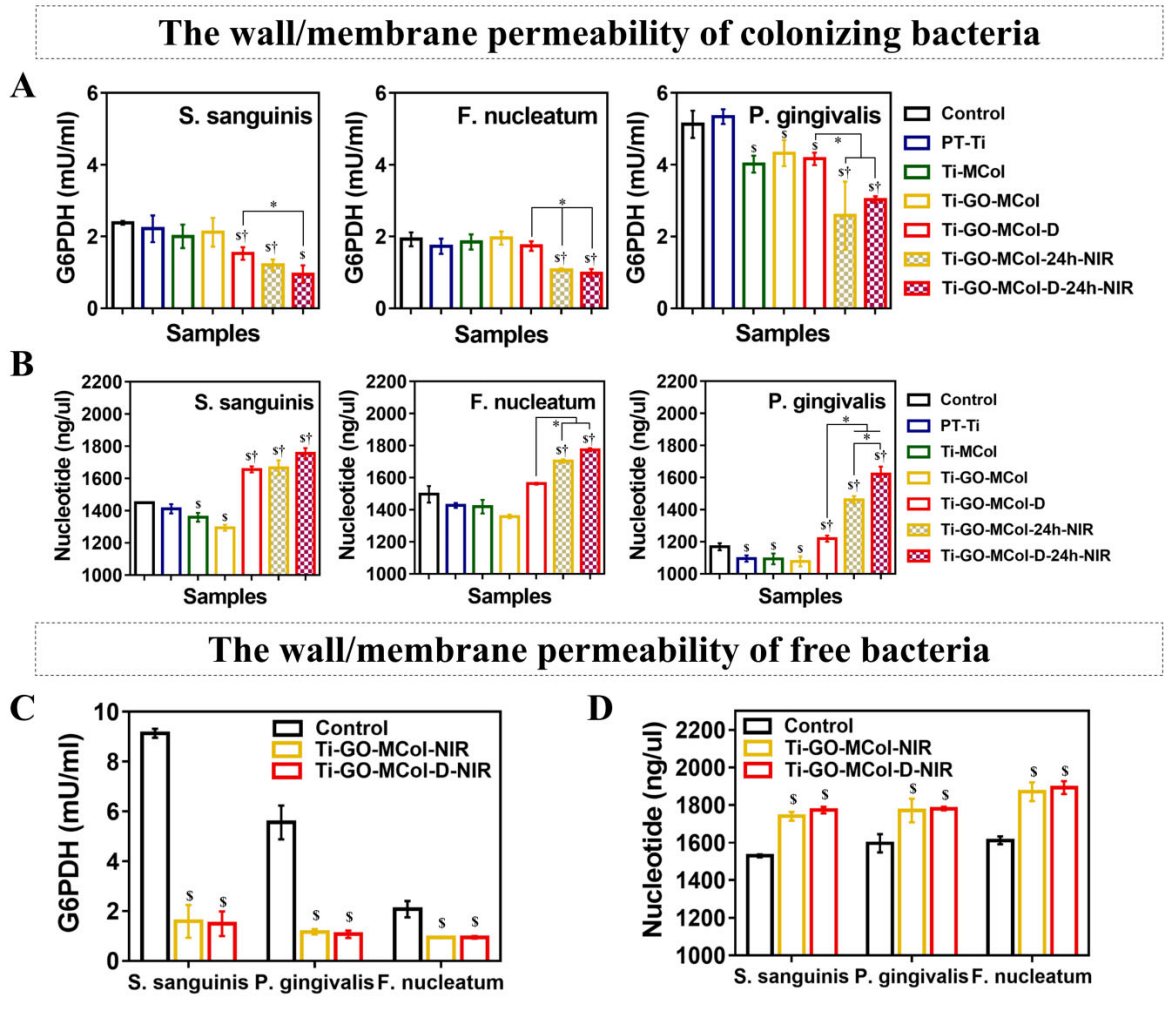
(**A–B**). Effect of modified coatings on the leakage G6PDH (A) and nucleic acid (B) from colonized bacteria regarding *S. sanguinis*, *F. nucleatum* and *P. gingivalis* before and after illumination. (**C–D**). Effect of modified coatings on the leakage G6PDH (C) and nucleic acid (D) from free bacteria regarding *S. sanguinis*, *F. nucleatum* and *P. gingivalis* before and after illumination. The tested G6PDH was collected from precipitation after centrifugation while nucleic was collected from supernatant after centrifugation. Error bars represent standard deviation of the mean for *n* = 3 (^$^*P* < 0.05 compared with control, ^†^*P* < 0.05 compared with Ti-GO-MCol, ^*^P < 0.05)

Additionally, the disorganization of the cell wall and disruption of the cell membrane in bacteria were noticed. These results motivated the further studies on the effect of modified coatings on the permeability of the bacterial wall/membrane. The levels of G6PDH and nucleotide were associated with the leakages of membrane [45]. Before illuminating, the levels of G6PDH in Ti-GO-MCol were similar with the levels of Ti-MCol, because GO was entrapped in the collagen leading to drop of direct-antibacterial activity (Fig. 6A). However, the enzyme levels of G6PDH in Ti-GO-MCol-D were lower than those of PT-Ti, indicating appreciable damages of bacterium wall and membrane at the presence of the DMADDM. This was because DMADDM is a kind of contact bactericidal quaternary ammonium salt. Its long-chain structure would pierce the bacterial cell wall, disturb the bacterial cell membrane and cause the leakage of contents, resulting in the death of bacteria [46].

Next, we further studied the potential of GO-based photothermal activity on damaging the bacterial wall/membrane and its synergistic effects with DMADDM. After illuminated, the levels of G6PDH in Ti-GO-MCol were significantly decreased, indicating a positive photothermal effect of GO on damaging the bacterial wall/membrane. Moreover, the combined use of DMADDM and illumination had a greater therapeutic efficacy against *S. sanguinis* compared with either treatment alone. Opposite trend has been found for nucleotide leakage, a direct indication of the disruption of bacteria membrane (Fig. 6B). A significant amount of 260 nm absorbing material has been observed in the Ti-GO-MCol-D, Ti-GO-MCol-24h-NIR and Ti-GO-MCol-D-24h-NIR, indicating a high-level leakage of nucleotides. It was noted that photothermal antibacterial GO exhibited a stronger antibacterial effect toward Gram-negative bacteria than Gram-positive bacteria. As for *S. sanguinis*, there was no significant difference between Ti-GO-MCol-D and Ti-GO-MCol-24h-NIR. However, for *F. nucleatum* and *P. gingivalis*, the levels of G6PDH and nucleotide in Ti-GO-MCol-24h-NIR were significantly lower and higher than those of Ti-GO-MCol-D, respectively. The flora causing peri-implant inflammation is mainly Gram-negative anaerobic bacteria [47], so it is self-evident that it has the advantage of good antibacterial ability. At the same time, *P. gingivalis* is one of the main pathogens causing periodontal disease [48]. Therefore, the coating prepared by us showed good antibacterial effect on *F. nucleatum* and *P. gingivalis*, which might have more advantages in inhibiting peri-implant inflammation and periodontal disease.

In the implant infection caused by bacteria, in addition to the colonized bacteria, free bacteria may also exist. Therefore, we further investigated the antibacterial effect of our modified coatings on free bacteria. Figure 6C and D showed the antibacterial ability of the coatings against free bacteria. When the bacterial solution was immediately illuminated, compared with the control group, the content of G6PDH in centrifugal precipitation of Ti-GO-MCol decreased significantly, indicating that the bacterial structure was destroyed. The increase of nucleotide concentration in the supernatant also supported this conclusion. Moreover, no notable changes in G6PDH and nucleotide were observed between Ti-GO-MCol-NIR and Ti-GO-MCol-D-NIR, indicating that DMADDM did not produce additional degree of facilitation. It was possible that the direct contact killing effect of DMADDM was not sufficient to damage free bacteria.

Overall, our modified coatings offered a unique combination of multiantibacterial mechanisms, including photothermal antibacterial activity and direct-antibacterial activity. On the one hand, the photothermal effect of GO would effectively damage the bacterial membrane and increase its permeability, leading to further permeation of inclusions of the bacteria. It is thought to not only be clearance of free Gram-positive and Gram-negative bacteria but also have a pronounced killing effect on bacterial colonization. On the other hand, the DMADDM directly disrupted the integrity of bacterial membrane and exerted a synergistic fungicidal effect.

### Cell response to modified coatings

#### HGF response to modified coatings

**Figure 7.**
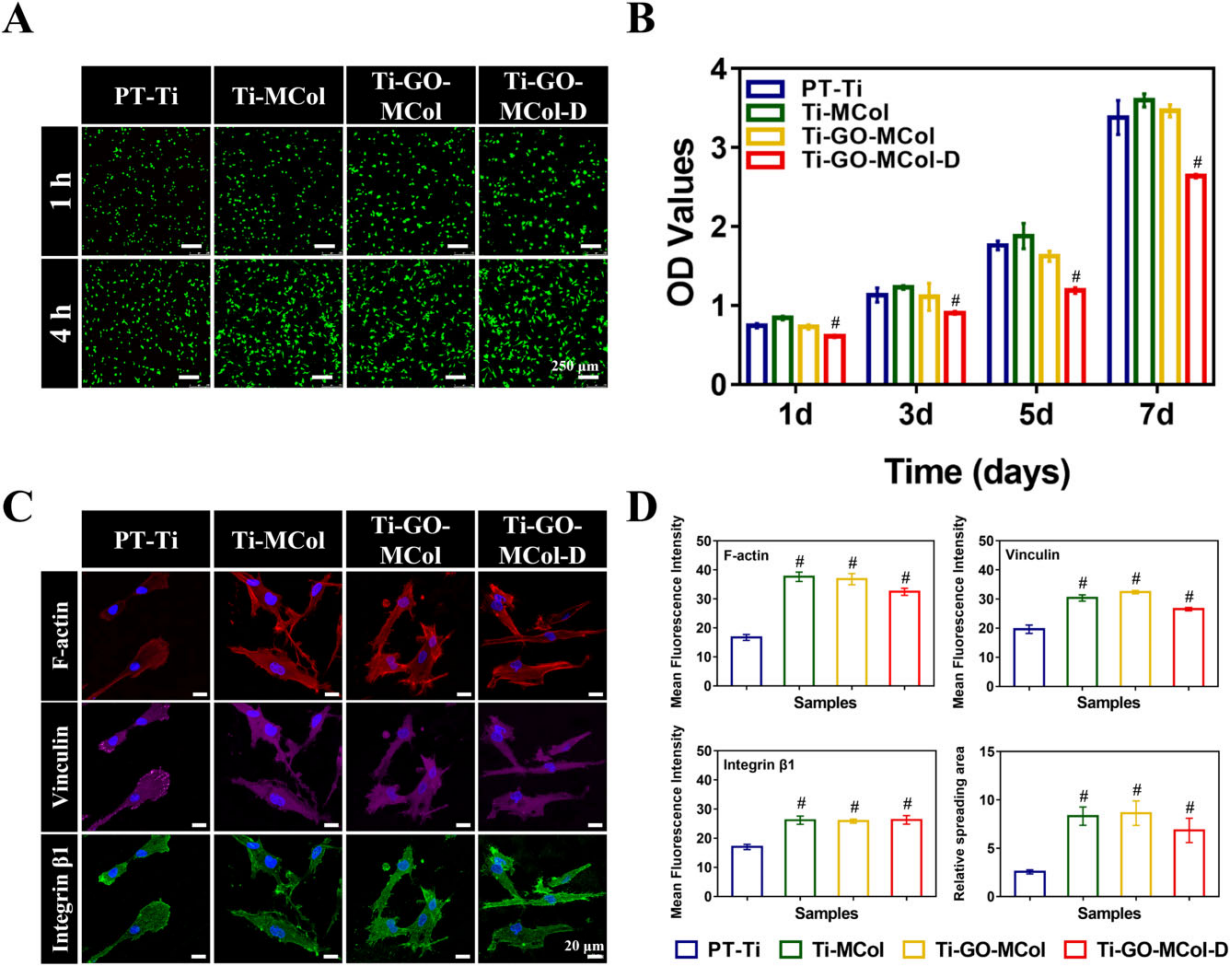
(**A**) The live/dead staining of HGFs on PT-Ti, Ti-MCol, Ti-GO-MCol and Ti-GO-MCol-D after cultured for 1 and 4 h. Scale bars represent 250 μm. (**B**) The proliferation of HGFs on PT-Ti, Ti-MCol, Ti-GO-MCol and Ti-GO-MCol-D surfaces for 1, 3, 5 and 7 days. (**C**) Immuno-fluorescence staining of adhesion-related proteins detected by confocal laser scanning microscopy on PT-Ti, Ti-MCol, Ti-GO-MCol and Ti-GO-MCol-D samples after incubation for 24 h. Scale bars represent 20 μm. (**D**) Quantification results of F-actin, vinculin, integrin β1 and relative cell spread area. Error bars represent standard deviation of the mean for *n* = 3 (^#^*P* < 0.05 compared with PT-Ti)

As for the antibacterial dental implant abutment, the biological function of the antibacterial coatings cannot be ignored, especially for soft tissue sealing. Hence, we examined the effects of modified coatings on HGF response, including cell proliferation, early adhesion and spread. Figure 7A showed that with the extension of culture time, the cell number increased for all the samples. However, there were some differences in cell number and morphology between modified coatings and unmodified coatings. Compared with PT-Ti, more cells were adhered and better spread was observed on the modified coatings, showing a typical long spindle shape. More importantly, the presence of GO displayed no significant influence on cell morphology and spreading, since our unique layered structure potently attenuated GO toxicity. Moreover, a CCK-8 assay was also performed to confirm the above observation (Fig. 7B). It was clear that by comparing Ti-MCol with Ti-GO-MCol, the presence of GO had no major effect on cell proliferation, further confirming that the coating with ‘sandwich’ structure was an efficient method to reduce the toxicity of GO. Meanwhile, the addition of DMADDM showed a decreased OD value, compared with Ti-MCol, probably due to the fact that DMADDM had deleterious effects on cell membrane stability. But the HGFs cultured on Ti-GO-MCol-D were obviously proliferating as the coincubation time was getting longer, suggesting that the effect of DMADDM on cells was within an acceptable range.

Understanding the adhered roles of HGF around percutaneous implants is of critical important for the promoted sealing ability. Therefore, we further investigated the expression of characteristic proteins related to adhesion, including F-actin, vinculin and integrin β1 (Fig. 7C). F-actin could reflect the cytoskeleton and adhesion plaques of cells. After incubated for 24 h, the F-actin of HGFs on PT-Ti exhibited round, and only a small number of short fibers gathered at the cell edge while the cells on the modified coatings showed long spindle spreading. The presence of mineralized collagen resulted in robust stress fibers distributed in the cytoplasm. Previous studies have been demonstrated that collagen was conductive to form stress fibers, which was in favor of establishment of tight cell–matrix connection and thus increased soft tissue integration [49]. Besides, the increased roughness and hydrophilicity that benefited from the formation of apatite with scattered distribution on collagen surface also facilitated cytoskeleton organization and cell adhesion. This was in agreement with other reports where micro-rough surface and hydrophilic surface could effectively increase extensive and tight soft tissue sealing [50]. More importantly, there was no indication that the addition of GO adversely affected cytoskeleton organization, probably due to the advantage of our ‘sandwich’ structure. However, in Ti-GO-MCol-D group, the distribution of cytoskeleton changed slightly, with shrinkage in the size, probably due to the damage of DMADDM to cell membrane.

HGFs adhered to natural teeth or materials were highly correlated with focal adhesion. Vinculin and integrin are essential to the formation of stable focal adhesions. Cytoskeleton organization was highly correlated with the intensity of vinculin and integrin, where well-organized actin cytoskeleton was followed by high intensity of vinculin and integrin. Quantification results from Image J further confirmed the above observation (Fig. 7D). According to the calculation results of Image J, the relative spreading area of HGFs on PT-Ti was about 2.5. It was found that the relative spreading area on Ti -MCol and Ti-GO-MCol was 8.3 and 8.6, respectively, which was more than three times as much as that on PT-Ti. The relative spreading area of HGFs on Ti-GO-MCol-D was slightly smaller, only about 6.8. But it was still about 2.7 times as much as that of PT-Ti. The relative spreading area of cell well correlated with the fluorescence intensity of F-actin, vinculin and integrin β1. The appearance of collagen layer also significantly increased the fluorescence intensity of F-actin, vinculin and integrin β1. More importantly, collagen layer could effectively reduce the GO toxicity. Compared with Ti-MCol, obvious changes in the fluorescence intensity levels of F-actin, vinculin and integrin β1 were not observed in the Ti-GO-MCol. When DMADDM was added, the fluorescence intensity of the Ti-GO-MCol-D decreased slightly. But it was still significantly higher than that of the control group. In conclusion, the positive enhancements of mineralized collagen modification on cell adhesion, cytoskeleton organization and focal adhesion were significant. Another advantage of collagen modification was the usage of collagen as the carrier for antibacterial components such as DMADDM. More importantly, our unique ‘sandwich’ coating structure was effective to reduce toxic effect of GO without compromising its photothermal effect [51, 52].

#### Effect of modified coatings on decreased photothermal damage for HGF response

**Figure 8.**
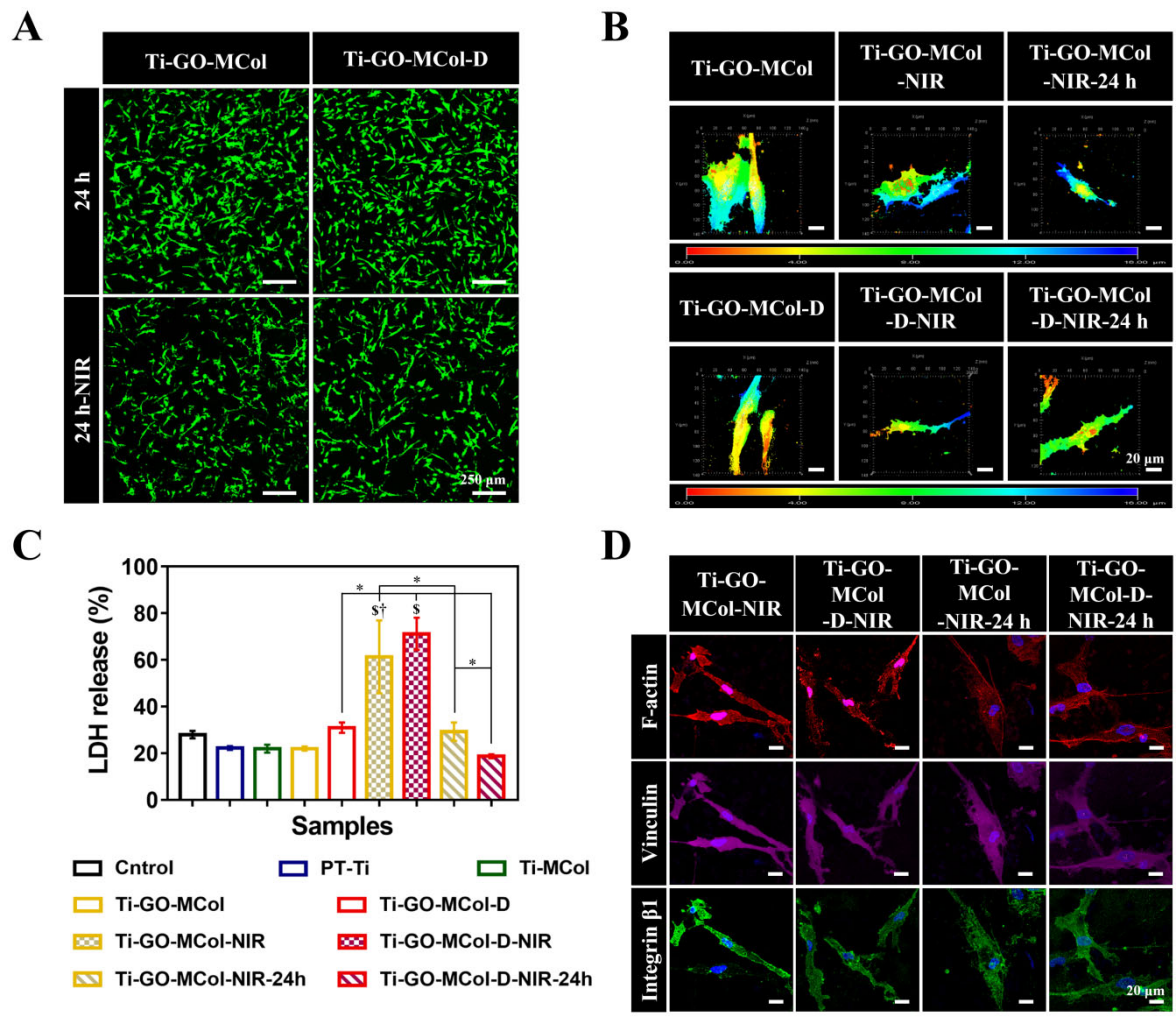
(**A**) The live/dead staining of HGFs on Ti-GO-MCol and Ti-GO-MCol-D before and after illumination after cultured for 24 h. Scale bars represent 250 μm. (**B**) The 3D confocal microscopy images of HGFs on Ti-GO-MCol and Ti-GO-MCol-D before and after illumination, as well as further culturing for one more period of 24 h. Scale bars represent 20 μm. (**C**) LDH activities in the medium after the contact between HGFs and different sample. Error bars represent standard deviation of the mean for *n* = 3 (^$^*P* < 0.05 compared with control, ^†^*P* < 0.05 compared with Ti-GO-MCol, ^*^P < 0.05). (**D**) Immuno-fluorescence staining of adhesion-related proteins of HGFs on Ti-GO-MCol and Ti-GO-MCol-D before and after illumination. NIR represents illumination for 15 min and NIR-24 h represents that the samples continued to culture for another 24 h after illumination. Scale bars represent 20 μm

The photothermal therapy is not bacteria-selective, they act on both bacteria and cells. In photothermal therapy, the temperature increases that may cause damage to the normal cells. We further investigated the photothermal effect on cell damage. Firstly, the adhesion state of cells on Ti-GO-MCol and Ti-GO-MCol-D after irradiating was examined (Fig. 8A). It was found that after culturing with Ti-GO-MCol and Ti-GO-MCol-D, the number of cells decreased slightly after illumination, and the cells still showed a clear long spindle type, indicating that HGFs still spread well after the illumination and the rise of temperature.

Then, we further examined whether the illumination would influence cell membrane. Fluorescent PKH26 staining followed by 3D confocal imaging was performed to evaluate the 3D orientation of HGFs on the modified coatings with or without illumination (Fig. 8B). The results indicated that the illumination indeed resulted in slight damage of cell membrane. Following illuminated treatment, membrane ruffles increased at the edge of the cells. However, after culturing for one more period of 24 h, cells on the Ti-GO-MCol and Ti-GO-MCol-D groups showed nearly normal cellular morphology similar to corresponding untreated group. LDH, which was used as an indication of the disruption of the HGF cell membrane, further demonstrated the above results. As it was shown in Fig. 8C, the LDH results showed that NIR light did have a certain impact on cell membrane, as evidenced by the fact that the LDH content of Ti-GO-MCol and Ti-GO-MCol-D in the supernatant increased after illumination. However, the LDH contents of Ti-GO-MCol and Ti-GO-MCol-D in the supernatant returned to the normal level, after culture for another 24 h [53]. The unique recovery ability of cell membrane was likely due to our ‘sandwich’ structure coating. Although GO inclusion in bilayers resulted in the loss of the direct-antimicrobial actions, this would definitely have a positive impact on biocompatibility. Moreover, the mineralized collagen layers played an important role in membrane recovery. Collagen is a kind of protein with high content in extracellular matrix (ECM), which can form reticular basement membrane with other ECM proteins such as laminin to provide the possibility for the repair of cell membrane and the restoration of permeability [54]. For instance, Huang *et al.* [55] demonstrated that collagen was beneficial in accelerating the tissue regeneration and repair of injured cells. In addition, apatite formed from mineralized collagen can further support cell growth and fibroblast metabolism and accelerate the self-repair of HGFs [56]. The results of Jiang *et al.* [57] showed that the deposition of hydroxyapatite (HA) markedly improved cell activity, probably since the bioactive HA would be able to bind mineralization-related proteins, leading to a favorable biomimetic microenvironment.

We also investigated the cytoskeleton (F-actin) and the related to adhesion characteristic proteins (vinculin and integrin β1) of HGFs after illuminating and further cultured for another 24 h on Ti-GO-MCol and Ti-GO-MCol-D (Fig. 8D). It can be observed from the results that HGFs on the surface of Ti-GO-MCol and Ti-GO-MCol-D were slightly damaged after illumination. After 24 h of culture, actin cytoskeletal remodeling, monitored by staining with red phalloidin, showed the beginnings of stress fiber formation. We also observed more intensity staining of focal adhesion-related proteins (vinculin and integrin β1) after switching to permissive temperature (37°C) for 24 h, indicating ongoing cell adhesion recovery. Cell adhesion to the matrix is vital for normal cell functioning and proper adhesion is thought to be prerequisite for optimal function of soft tissue sealing. The above results indicated that the temperature rise caused by light may do slight harm to cells, but due to the good microenvironment provided by mineralized collagen and the self-repair ability of cells, this damage was within an acceptable range.

### Effect of modified coatings on the balance between oral bacteria and HGFs

**Figure 9.**
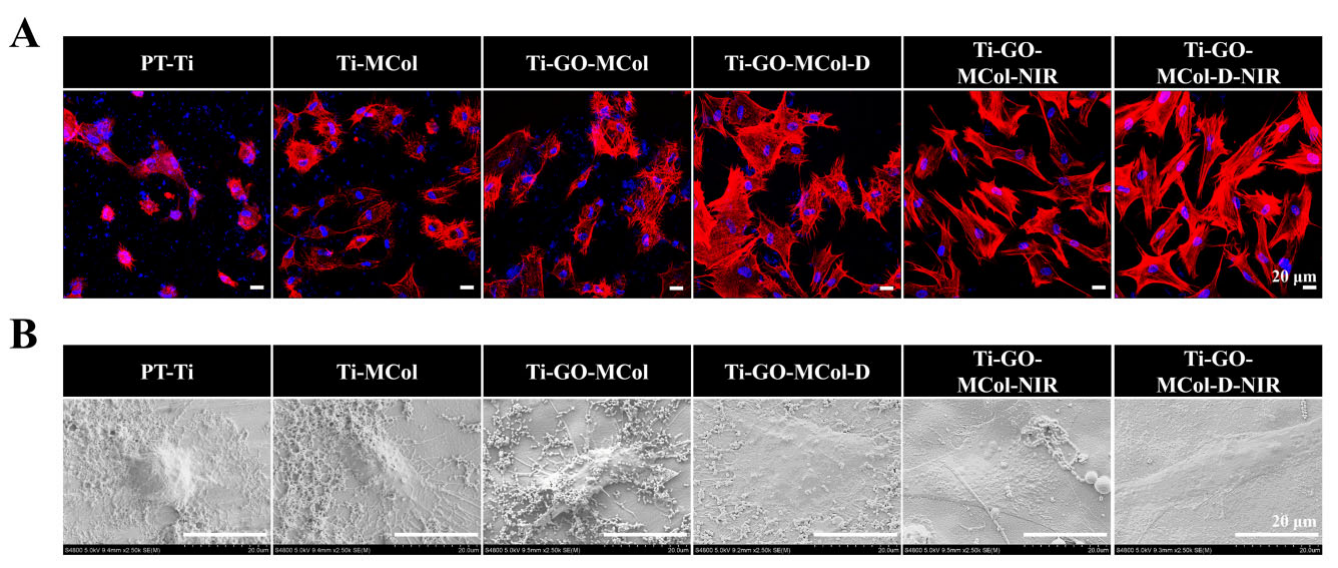
(**A**) F-actin staining images of HGFs after cocultured with *S. sanguinis* for 4 h on different samples. Scale bars represent 20 μm. (**B**) SEM images of the coculture. Scale bars represent 20 μm

In natural oral environments, bacteria would compete against neighboring cells for the colonization of the dental implant abutment [58]. Once bacteria win the competition, tissue cell function will be impaired, and the risk of the implant failure will be increased. Thus, if we want to gasp the boundary of antibacterial ability based on excellent bioactive function, it was imperative to analyze simultaneously their antibacterial and biocompatibility properties under the condition of facultative anaerobic bacteria *S. sanguinis* and HGFs coculture. As shown in Fig. 9A, the presence of bacteria reduced the coverage by HGF cells of the implant materials after 4 h of growth on PT-Ti. The addition of collagen had a positive role in regulating the actin cytoskeleton, indicating that the importance of the mineralized collagen layer. However, the bacteria were present around the cells, indicating the existence of the interference of bacteria. It was noted that with the advent of antibacterial activity, the adhered cell number increased, which was accompanied by the increased formation of stress fiber. It was not surprised that the best results were obtained for Ti-GO-MCol-D-NIR. Robust antibacterial ability benefited cell competition against bacteria. The SEM results shown in Fig. 9B were also consistent with the confocal results. A large number of bacterial aggregations could be observed on PT-Ti, Ti-MCol and Ti-GO-MCol while the number of visible bacteria from Ti-GO-MCol-D, Ti-GO-MCol-NIR and Ti-GO-MCol-D-NIR had decreased substantially. NIR light exhibited a better antibacterial effect than DMADDM. Moreover, optimal reduction of bacteria was achieved in the combined use of DMADDM and NIR light. Of note, NIR light caused distinct damage to the bacterial cell membrane, but the cells still displayed a flat spreading morphology adherent to the substrate. In the ‘race for the surface’, our modified coatings would help cells win this race. Inspired by ‘overdone is worse than undone’, our modified coatings were partly sacrificed on its antibacterial ability, but obtained excellent bioactivity. Moreover, our unique ‘sandwich’ structure and mineralized collagen can further increase cell viability, which might be strengthened to resist bacterial infections. Overall, we grasped the boundary of antibacterial property on biological activity where HGFs were superior to bacteria with respect to the competitive colonization on our modified coatings. Taken together, the balance of antibacterial efficacy and avoiding side effect on cell toxicity has been a challenging task for dental implant abutment applications. In this study, Ti-GO-MCol with sandwich structure was constructed successfully via layer-by-layer technology to achieve this balance. Although the excellent antibacterial efficacy of the Ti-GO-MCol with the long term and recyclable use was not surprising due to the photothermal antibacterial activity, the samples also demonstrated superior HGF cell response. The HGF excellent response of the coatings depends on our specified ‘sandwich’ structure and mineralized collagen of top layer. More importantly, the *invitro* coculture study further suggested that our modified coatings were the preferred materials with respect to soft tissue sealing under conditions of pathogen challenge. Moreover, the collagen of top layer can be used as an efficient carrier for DMADDM, leading to further enhanced antibacterial ability and providing effective alternatives for multimode of antibacterial.

Overall, our technologies would be applied into surface modification for dental implant abutment in terms of soft tissue sealing. This simple but effective approach that mimics ‘sandwich’ structure would grasp the boundary of antibacterial better for the sealing of soft tissue. Meanwhile, remotely controllable and circulating antibacterial ability is beneficial for promising practical treatment of dental implant reinfection, without additional surgery or treatment, which is particularly advantageous for clinical application.

## Conclusion

In this study, we successfully synthesized a GO-decorated Ti dental implant abutment through layer-by-layer method with the assistance of PDA. The coating with ‘sandwich’ structure possessed suitable antibacterial capability and exerted superior cytocompatibility. The obtained results demonstrated that our coatings showed antibacterial activity against both Gram-positive and Gram-negative bacteria. Moreover, collagen located on the outermost coatings would additionally incorporate DMADDM. Light-assisted photothermal therapy combined with DMADDM showed the best synergistic antibacterial effect, and this effect was retained after five cycles. Cytocompatibility of GO-modified coatings was enhanced according to the assessments of cell adhesion, cytoskeleton organization and proliferation effects, probably due to our quite sandwich structure and mineralized collagen layer. More importantly, the mineralized collagen layer had a critical role in soft tissue, which was involved in protecting the tissue cells from damage caused by photothermal antibacterial therapy. The results of cell bacterial coculture experiment further demonstrated that our coating provided the best opportunities for soft tissue sealing to form on bacterially contaminated implant abutment surfaces.

## Supplementary data

Supplementary data are available at *REGBIO* online.

## Supplementary Material

rbac024_Supplementary_Data
