# Supplementary material for: Graphene oxide and mineralized collagen-functionalized dental implant abutment with effective soft tissue seal and romotely repeatable photodisinfection
Source: Regen Biomater. 2022 Apr 29;9:rbac024. doi: 10.1093/rb/rbac024 (PMC9071057; doi:10.1093/rb/rbac024)
Supplement: rbac024_Supplementary_Data [file rbac024_supplementary_data.docx]

**Supplementary information**

Graphene oxide and mineralized collagen-functionalized dental implant abutment with effective soft tissue seal and romotely repeatable photodisinfection

Yichun Gao, Ke Kang, Bin Luo, Xiaoqing Sun, Fang Lan, Jing He^*^ and Yao Wu^*^

*National Engineering Research Center for Biomaterials, Sichuan University, Chengdu 610064, P. R. China*

* To whom correspondence should be directed

E-mail: [jinghe30@scu.edu.cn](mailto:jinghe30@scu.edu.cn) ; [wuyao@scu.edu.cn](mailto:wuyao@scu.edu.cn)





**Fig**ure **S1.** FTIR spectrum of Ti-GO-Col-D, and Ti-MCol.


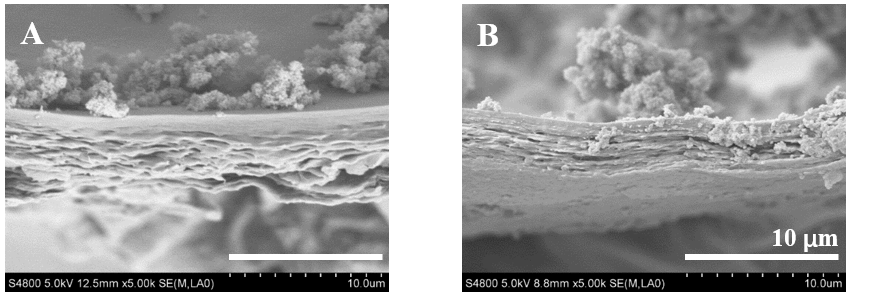


**Figure S2.** SEM images of cross section of (a). Ti-GO-MCol and (b). Ti-GO-MCol-D.

**Table 1.** The surface roughness of PT-Ti, Ti-MCol, Ti-GO-MCol and Ti-GO-MCol-D.

| **Samples** | **Rq (nm)** |
| --- | --- |
| PT-Ti | 45.37±2.55 |
| Ti-MCol | 93.51±5.69 |
| Ti-GO-MCol | 97.13±1.36 |
| Ti-GO-MCol-D | 95.99±2.71 |


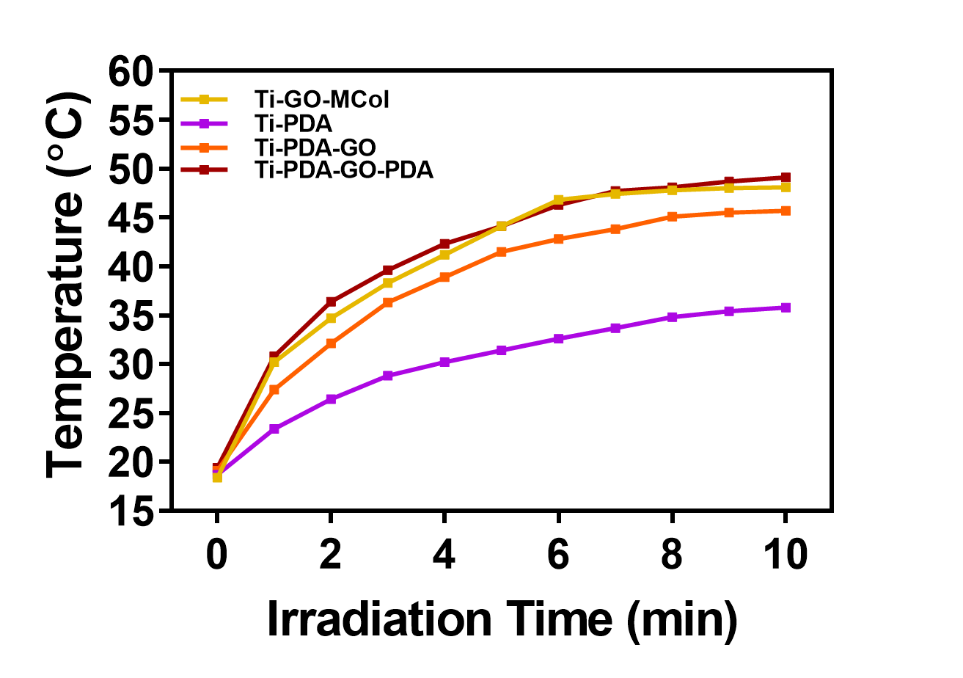


**Fig**ure **S3.** Photothermal curves of Ti-GO-MCol, Ti-PDA, Ti-PDA-GO and Ti-PDA-GO-PDA under laser irradiation (808 nm, 1.0 W/cm^2^).


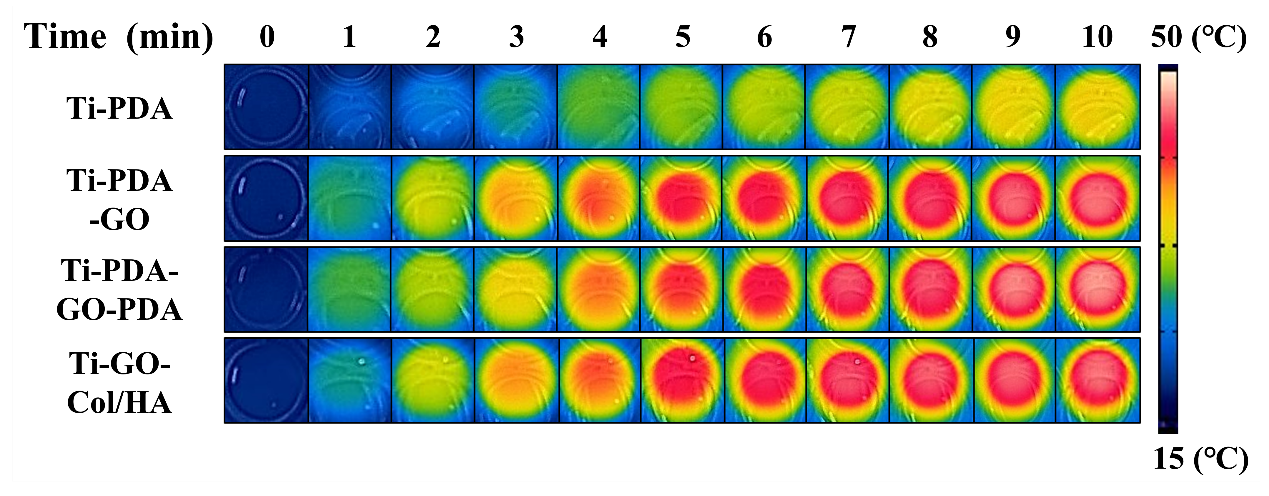


**Fig**ure **S4.** Infrared thermal images of Ti-PDA, Ti-PDA-GO, Ti-PDA-GO-PDA, and Ti-GO-MCol (808 nm, 1.0 W/cm^2^).


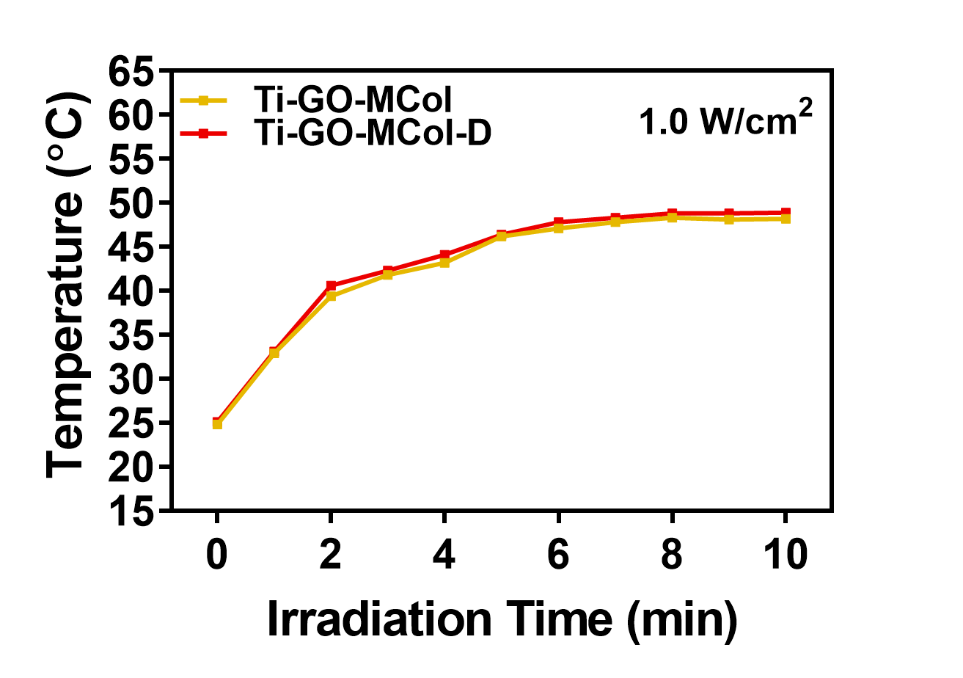


**Fig**ure **S5.** Photothermal curves of Ti-GO-MCol and Ti-GO-MCol-D after immusered in PBS for 10 days (808 nm, 1.0 W/cm^2^).
